# The Inhibitory Effect of Ojeoksan on Early and Advanced Atherosclerosis

**DOI:** 10.3390/nu10091256

**Published:** 2018-09-06

**Authors:** Byung Hyuk Han, Chang Seob Seo, Jung Joo Yoon, Hye Yoom Kim, You Mee Ahn, So Young Eun, Mi Hyeon Hong, Jae Geon Lee, Hyeun Kyoo Shin, Ho Sub Lee, Yun Jung Lee, Dae Gill Kang

**Affiliations:** 1 Hanbang Cardio-Renal Syndrome Research Center, Wonkwang University, 460, Iksan-daero, Iksan, Jeonbuk 54538, Korea; arum0924@nate.com (B.H.H.); mora16@naver.com (J.J.Y.); hyeyoomc@naver.com (H.Y.K.); aum2668@naver.com (Y.M.A.); eunsoyg@wku.ac.kr (S.Y.E.); mihyeon123@naver.com (M.H.H.); john1027@snu.ac.kr (J.G.L.); host@wku.ac.kr (H.S.L.); 2 College of Oriental Medicine and Professional Graduate School of Oriental Medicine, Wonkwang University, 460, Iksan-daero, Iksan, Jeonbuk 54538, Korea; 3 K-herb Research Center, Korea Institute of Oriental Medicine, 1672 Yuseong-daero, Yuseong-gu, Daejeon 34054, Korea; csseo0914@kiom.re.kr (C.S.S.); hkshin@kiom.re.kr (H.K.S.); 4 Department of Physiology, Seoul National University College of Medicine, 103, Daehak-ro, Jongno-gu, Seoul 03080, Korea; 5 Department of Dental Pharmacology, School of Dentistry and Institute of Oral Bioscience, BK21 Plus, Chonbuk National University, 567 Baekje-daero, Jeonju, Jeonbuk 54896, Korea

**Keywords:** Ojeoksan, atherosclerosis, vascular inflammation, vasodilation, hypertension, adhesion molecule

## Abstract

Atherosclerosis is closely related to vascular dysfunction and hypertension. Ojeoksan (OJS), originally recorded in an ancient Korean medicinal book named “Donguibogam”, is a well-known, blended herbal formula. This study was carried out to investigate the beneficial effects of OJS on atherosclerosis in vitro and in vivo. Western-diet-fed apolipoprotein-E gene-deficient mice (ApoE −/−) were used for this study for 16 weeks, and their vascular dysfunction and inflammation were analyzed. OJS-treated ApoE −/− mice showed lowered blood pressure and glucose levels. The levels of metabolic parameters with hyperlipidemia attenuated following OJS administration. Hematoxylin and eosin (H&E) staining revealed that treatment with OJS reduced atherosclerotic lesions. OJS also suppressed the expression of adhesion molecules and matrix metalloproteinases (MMPs) compared to Western-diet-fed ApoE −/− mice and tumor necrosis factor-alpha (TNF-α)-stimulated human umbilical vein endothelial cells (HUVECs). Expression levels of MicroRNAs (miRNA)-10a, -126 3p were increased in OJS-fed ApoE −/− mice. OJS significantly increased the phosphorylation of endothelial nitric oxide synthase (eNOS) and protein kinase B (Akt), which are involved in nitric oxide (NO) production. OJS also regulated eNOS coupling by increasing the expression of endothelial GTP Cyclohydrolase-1 (GTPCH). Taken together, OJS has a protective effect on vascular inflammation via eNOS coupling-mediated NO production and might be a potential therapeutic agent for both early and advanced atherosclerosis.

## 1. Introduction

Inflammatory disorder is commonly known to be a major cause of Atherosclerosis [1] and characterized as a chronic inflammatory disease of the arterial wall [2]. The main reasons of early stage of atherosclerosis are hyperglycemia, high blood serum triglyceride levels, low concentration of high-density lipoprotein (HDL) cholesterol, high blood pressure, and central obesity [3]. Acetylcholine (ACh)-induced endothelium-dependent vasorelaxation is mediated by nitric oxide (NO), which acts via soluble guanylyl cyclase and cyclic Guanosine monophosphate (GMP). Endothelial dysfunction is a key marker of early stage of atherosclerosis. This process is mainly regulated by the expressions of cellular adhesion molecules on the endothelium. 

The expression of adhesion molecules increases in atherosclerotic lesion sites and can be the cause for vascular dysfunction and advanced atherosclerosis [4]. Mediated signaling by cytokines, such as tumor necrosis factor alpha (TNF-α), induce the expression levels of cell adhesion molecules, which is an important mediator in the pre-inflammatory process [5]. MicroRNAs (miRNAs) are single-stranded, non-coding, small RNAs that regulate gene expression by destabilizing target mRNAs and/or inhibiting translation. For example, miR-126 was well known to reduce the expression of adhesion molecules, such as intracellular adhesion molecule-1 (ICAM-1), vascular cell adhesion molecule-1 (VCAM-1), and endothelial cell selectin (E-selectin), by directly targeting the 3′ untranslated region (3′UTR) of these genes [6,7]. In addition, micro RNA-10a (MiR-10a) regulated the expression of Mitogen-Activated Protein Kinase Kinase Kkinase 7 (MAP3K7) and Transforming growth factor beta-activated kinase 1 (TAK1), and both of which regulate Inhibitor of NF-kappa B (IκB) degradation [8]. Adhesion molecules such as ICAM-1 and VCAM-1 play key roles in the process of atherosclerosis. Endothelial dysfunction is thought to be the cause of the development of atherosclerosis [9]. All vascular cells including endothelial cells and macrophages secrete matrix metalloproteinases (MMPs), especially MMP-2 and MMP-9, which are synthesized in atheroma plaques, and are particularly prevalent in rupture-prone shoulder regions [10]. MMPs degrade collagen and allow for smooth-muscle cell migration within a vessel. Moreover, this begets an accumulation of other cellular material, resulting in atherosclerosis and ischemic events to tissues [11].

Endothelial NO synthase (eNOS) produces NO, which is a key regulator in the vasodilation process. NO regulates early stages of inflammatory processes by downregulating nuclear factor Nuclear factor-kappa B (NF-κB) [12,13]. Cardiovascular disease has a variety of causes, involving uncoupled eNOS signaling [14]. Studies with endothelial cells and isolated vessels supported the important role of eNOS coupling, showing that the inhibition of GTP cyclohydrolase-1 (GTPCH) results in reduced NO synthesis. In vascular disorders such as atherosclerosis, NO activity is decreased, whereas oxidative stress is upregulated and results in endothelial dysfunction. Enzymatic eNOS coupling by the cofactor tetrahydrobiopterin (BH4) plays a key role in maintaining endothelial function [15]. In the case of cardiovascular risk, the availability of BH4 decreased in the vessel wall with subsequent eNOS dysfunction may be a crucial determinant of impaired NO-mediated endothelial function. Protein kinase B (Akt) can activate eNOS, which leads to NO production. Additionally, the Phosphoinositide 3-kinase (PI3K)/Akt/eNOS pathway plays a key role in NO production [16,17]. 

Ojeoksan (OJS; Wuji-san in Chinese, Goshaku-san in Japanese) is composed of 17 herbal medicines (Table 1). OJS has been documented in a traditional Korean medical book named “Donguibogam” and in a traditional Chinese medical book named “Tae-Pyung-Hye-Min-Hwa-Je-Guk-Bang”. According to records, OJS has been used as a pain-relieving agent and used to manage rash caused by circulation disadvantage of qi (氣) and blood (血), food (食), cold (寒), and congestion (痰). This study focused on the effects of OJS on blood (血) circulation disorders. In Korea, OJS is ranked first in terms of oriental health treatment medicated days and medical expenses, as it is included in 56 prescription drugs [18]. A previous study has determined whether OJS can alleviate liver inflammation induced by liver toxicity [19]. However, there are no reports regarding the protective effects of OJS against cardiovascular disease. Apolipoprotein-E gene-deficient mice (ApoE −/− mice) have been shown to develop hyperlipidemia and atherosclerosis as well as hypertension and endothelial dysfunctions [20]. Therefore, the present study was carried out to investigate whether OJS has a protective role in vascular inflammation and suppresses atherosclerotic lesions in ApoE −/− mice.

## 2. Material and Methods

### 2.1. High-Performance Liquid Chromatography (HPLC) Analysis of OJS

Chemical marker compounds in an OJS sample were analyzed using a Shimadzu Prominence LC-20A series (Shimadzu Co., Kyoto, Japan) equipped with a solvent delivery unit (LC-20AT), an online degasser (DGU-20A3), a column oven (CTO-20A), an auto sample injector (SIL-20AC), and a photodiode array (PDA) detector (SPD-M20A). The chromatographic data were acquired and processed using LC solution software (Version 1.24, SP1, Kyoto, Japan). All marker compounds were separated on a Phenomenex (Phenomenex, Torrance, CA, USA) Gemini C18 (250 × 4.6 mm, 5 μm), and the column oven was maintained at 40 °C. The mobile phases consisted of distilled water (A) and acetonitrile (J.T. Baker, Phillipsburg, NJ, USA) (B), both with 1.0% (*v*/*v*) acetic acid (Sigma-Aldrich, St. Louis, MO, USA). The gradient elution conditions were as follows: 15–25% B (0–20 min), 25–55% B (20–40 min), 55–100% B (40–45 min), 100% B (45–50 min), and 10–15% B (50–55 min). The re-equilibrium time was 15 min. The flow rate was maintained at 1.0 mL/min, and the injection volume was 10 mL.

### 2.2. Experimental Animals

Six-week-old male apolipoprotein-E gene-deficient mice (ApoE −/−) and normal C57BL6 mice were obtained from Saeronbio. Inc. (Uiwang, Korea) and then housed in cages with automatic temperature, humidity (22 ± 2 °C, 50–60%), and lighting (12 h light/dark cycle) conditions. They were fed a pelletized commercial chow diet for acclimatization for 2 weeks on arrival. After acclimatization, the animals were randomly divided into five groups: (1) the control (C57BL6 mice + regular diet + Distilled Water (DW), *n* = 15); (2) ApoE −/− (ApoE −/− + Western diet + DW, *n* = 13); (3) telmisartan (ApoE −/− + Western diet + telmisartan 1 mg/kg/day, *n* = 13); (4) OJS low (ApoE −/− + Western diet + OJS 50 mg/kg/day, *n* = 14); and (5) OJS high groups (ApoE −/− + Western diet + OJS 200 mg/kg/day, *n* = 14). The angiotensin receptor blocker, telmisartan, an anti-atherosclerotic agent, was chosen as a positive control [21]. A Western diet was also carried out for 16 weeks to sufficiently induce atherosclerosis in the experimental animals [22]. The control group received a regular diet (RD) (D12450B) and ApoE −/− groups received a Western diet (WTD) (D12079B) (Table 2), respectively, for 16 weeks by oral intake. The RD and WTD were purchased from Saeronbio. Inc. (Uiwang, Korea). At the end of the experimental period, all mice were sacrificed after 12 h fasting, and blood samples were collected into 1 mg/mL ethylendiaminetetraacetic acid (EDTA)-coated tube after pulled out the eyeball. All procedures were approved by the animal Ethics Committee (WKU12-15) and conformed to the guidelines specified by the country.

### 2.3. Cell Cultures

Human Umbilical Vein Endothelial Cells (HUVECs) were purchased from American Type Culture Collection (ATCC) (CRL-2873; Manassas, VA, USA). HUVECs were cultured at a density of 5 × 10^5^ cells/mL in Roswell Park Memorial Institute (RPMI) supplemented with 10% fetal bovine serum and 100 U/mL penicillin G and were then incubated at 37 °C in a humidified atmosphere containing 5% CO_2_ and 95% air.

### 2.4. Measurement of Systolic Blood Pressure

Systolic blood pressure (SBP) was measured by using the noninvasive tail-cuff plethysmography method and was recorded by using an automatic sphygmomanometer (MK2000; Muromachi Kikai, Tokyo, Japan). The systolic blood pressure (SBP) was measured at Weeks 4, 8, 12, and 16. The blood pressure of 7 mice per group was measured. At least five measurements were obtained at every session, and the mean of the five values within 5 mmHg was taken as the SBP level. Values are presented as the mean ± SEM of five measurements.

### 2.5. Plasma Biochemical Analysis

Blood glucose was measured from tail vein samples of whole blood using the One-touch ultra-blood glucose meter and a test strip (Life Scan Inc., Milpitas, CA, USA) at every 4 weeks. Approximately 500 μL of blood samples were collected from the periorbital vein for biochemical analysis per each mouse. Plasma glucose levels were measured using a commercial mouse ELISA kit (Abcam., Cambridge, MA, USA). Total cholesterol (T-cho), low-density lipoprotein-cholesterol (LDL-cho), very low-density lipoprotein-cholesterol (VLDL-cho), and high-density lipoprotein cholesterol (HDL-cho) levels in plasma were measured using commercially available kits (Abcam., Cambridge, MA, USA). Triglyceride (TG) level was measured using a commercial kit (AM157S-K, Asanpharm. Yeongcheon, Korea). The atherogenic index was calculated as follows: (Total cholesterol − HDL cholesterol)/HDL cholesterol [23].

### 2.6. Preparation of Carotid Artery Samples and Measurement of Vascular Reactivity

The carotid arteries of the mice were rapidly and carefully isolated and placed in cold Kreb’s solution of the following composition (mM): NaCl 118, KCl 4.7, MgSO_4_ 1.1, KH_2_PO_4_ 1.2, CaCl_2_ 1.5, NaHCO_3_ 25, glucose 10, and pH 7.4 (Sigma-aldrich, Saint Louis, MO, USA). The carotid arteries were removed from connective tissue and fat and cut into rings of 3 mm lengths. The carotid artery rings were suspended by means of two l-shaped stainless-steel wires inserted into the lumen in a tissue bath containing Kreb’s solution at 37 °C and aerated with 95% O_2_ and 5% CO_2_. The isometric forces of the rings were measured using a Grass FT 03 force displacement transducer connected to a Model 7E polygraph recording system (Grass Technologies, Quincy, MA, USA). In the carotid artery rings of mice, a passive stretch of 1 g was determined to be the optimal tension for maximal responsiveness to phenylephrine (10^−6^ M) (Sigma-aldrich, Saint Louis, MO, USA). The preparations were allowed to equilibrate for approximately 1 h with replacement of Kreb’s solution every 10 min. The relaxant effects of acetylcholine (ACh, 10^−10^–10^−6^ M) and sodium nitroprusside (SNP, 10^−11^–10^−7^ M) were studied in carotid artery rings constricted with phenylephrine (PE, 10^−7^ M).

### 2.7. Histopathological Staining of Aorta

Aortic tissues were fixed by using 10% (*v*/*v*) formalin (Junsei Chemical, Tokyo, Japan) in 0.01 M phosphate-buffered saline (PBS) (Gibco, Carlsbad, CA, USA) for 2 days with change of formalin solution every day to remove traces of blood from tissue. The tissue samples were dehydrated and embedded in paraffin (Leica, Wetzlar, Germany), and thin sections (6 μm) of the aortic arch from each group were then cut and stained with hematoxylin and eosin (H&E) (Sigma-Aldrich, St. Louis, MO, USA). For quantitative histopathological comparisons, each section was evaluated by Axiovision 4 Imaging/Archiving software (Axiovision 4, Carl Zeiss, Jena, Germany). Images were analyzed using the ImageJ (NIH, Bethesda, MD, USA) program to select and quantify H&E-stained areas as a fold of the total area of each image.

### 2.8. Measurement of Atherosclerotic Lesions by Oil Red O Staining

The thoracic/abdominal aorta were stained with Oil Red O to visualize neutral lipid (cholesteryl ester and triglycerides) accumulation. The inner aortic surface was stained with Oil Red O After rinsing with 60% isopropyl alcohol (Amresco, Solon, OH, USA) and distilled water, and images of Oil Red O-stained aortas were obtained with Axiovision 4 Imaging/Archiving software (Axiovision 4, Carl Zeiss, Jena, Germany). Images were analyzed using the ImageJ (NIH, Bethesda, MD, USA) program to select and quantify H&E-stained areas as a fold of the total area of each image.

### 2.9. Immunofluorescence

Frozen sections for immunofluorescence staining were placed on poly-l-lysine-coated slides (Fisher scientific, Pittsburgh, PA, USA). The slides were incubated with primary antibodies for ICAM-1, VCAM-1, and E-selectin (1:500; Santa Cruz, CA, USA) in humidified chambers for 1 h at room temperature in PBS followed by heat-induced (pressure cooker) sodium citrate antigen retrieval and then exposure to a 1:500 dilution of Alexa Fluor 594 secondary antibody (Life technology, Carlsbad, CA, USA). Finally, the slides were washed three times with PBS and cover slips were mounted with Dako fluorescent mounting medium onto glass slides that were examined under a fluorescence microscope (Nikon Eclipse Ti, Tokyo, Japan).

### 2.10. Western Blot Analysis

Aortic tissue and cell homogenates (protein of 30–50 μg) were separated using 10% SDS-polyacrylamide gel electrophoresis (PAGE) and transferred onto nitrocellulose membranes. Blots were then blocked by 5% Bovine Serum Albumin (BSA) (GenDEPOT, Katy, TX, USA) powder in Tris-bufferd saline (TBS) for 1 h, and incubated with the antibodies against ICAM-1, VCAM-1, E-selectin, MMP-2, MMP-9, eNOS, p-eNOS, Akt, p-AKT, and GTPCH (Santa Cruz Biotechnology, INC, Dallas, TX, USA) (1:1000 dilution in 0.05%TBS-T (Tween 20)). Subsequently, the membrane was then incubated with a secondary antibody of goat anti rabbit IgG or goat anti mouse IgG conjugated to horseradish peroxidase (Enzo Life Sciences, Farmingdale, NY, USA) (1:5000 dilution in 0.05%TBS-T), and the bands were detected with EzWestLumi plus solution (Cat. no. WSE-7120, Atto Corporation) using a ChemiDoc (Bio-Rad Laboratories, Hercules, CA, USA). Densitometry analysis of protein bands was conducted with the ImageJ (NIH, Bethesda, MD, USA) program.

### 2.11. RNA Preparation and Quantitative Real-Time Reverse Transcription-PCR (Real-Time RT-qPCR)

Total RNA was extracted from the aorta using Ribozol reagent (Amresco, Solon, OH, USA) according to the manufacturer’s instruction. The RNA quality was assessed by measuring the ratio of 260/280 nm light with a UV-spectrophotometer (Biophotometer plus, Eppendorf, Hambrug, Germany). The cDNA was synthesized from 500 ng mRNA via a 20 μL reverse transcription reaction incubated in the SimpliAmp Thermal Cycler (Life technology, Carlsbad, CA, USA). The sequences of primers and probes were as follows: ICAM-1 (forward: 5′-CTCACCCGTGTACTGGACTC-3′, reverse: 5′-CGCCGGAAAGCTGTAGATGG-3′), VCAM-1 (forward: 5′-ATGCCTGGGAAGATGGTCGTGA-3′, reverse: 5′-TGGAGCTGGTAGACCCTCGCTG-3′), E-selectin (forward: 5′-ATCATCCTGCAACTTCACC-3′, reverse: 5′-ACACCTCACCAAACCCTTC-3′), and Glyceraldehyde 3-phosphate dehydrogenase (GAPDH) (forward: 5′-CAAGGCTGAGAATGGGAAGC-3′, reverse: 5′-AGCATGTGGGAACTCAGATC-3′). The real-time RT-qPCR was carried out with an SYBR Green PCR Master Mix (Enzynomics, Inc., Daejeon, Korea) and performed at an initial denaturation step at 95 °C for 10 min, followed by 40 cycles at 95 °C for 15 s, and finally 60 °C for 60 s in the Step-One™ Real-Time PCR System (Applied Biosystems, Foster City, CA, USA). miR-10a and miR-126 3p were measured by using a hsa-mir-10a Real-Time RT-PCR detection kit (CPK1014) and a hsa-mir-126 3p Real-Time RT-PCR detection kit (CPK1083) (Cohesion Biosciences., London, UK). 

### 2.12. Measurement of NO Production Using Griess Reagent System

NO production in the culture supernatant was spectrophotometrically evaluated by measuring nitrite content, an oxidative product of NO. Nitrite levels was determined with the Griess Reagent solution (Promega, Madison, WI, USA) and proceeded according to the description of the manufacturer. The fluorescent intensity was then measured using a spectrofluorometer (Infinite F200 pro, Tecan, Switzerland) at an excitation and emission wavelength of 485 and 535 nm.

### 2.13. Fluorescence Microscopy

To examine intracellular NO generation, the Diaminofluorescein-2 Diacetate (DAF-2DA) (Merck Biosciences, Schwalbach, Germany) was used. In brief, HUVECs were cultured at 6-well plate. The cells were serum-starved for 8 h before incubation with treatments OJS. The HUVECs were then incubated with OJS for 30 min. DAF-2DA was added for the final 30 min of incubation. Reactions were stopped and fixed the cells by using 2% paraformaldehyde for 30 min at room temperature. Coverslips were examined with a fluorescence microscope equipped with an excitation filter (485–535 nm).

### 2.14. Statistical Analysis

All experiments were repeated at least three times. Statistical analyses were performed using *t*-tests. The results are expressed as mean ± standard error (S.E.), and the data were analyzed using one-way analysis of variance followed by a Student’s *t*-test to determine any significant differences. *p* < 0.05 indicated statistical significance.

## 3. Results

### 3.1. HPLC Analysis of OJS

**Figure 1:**
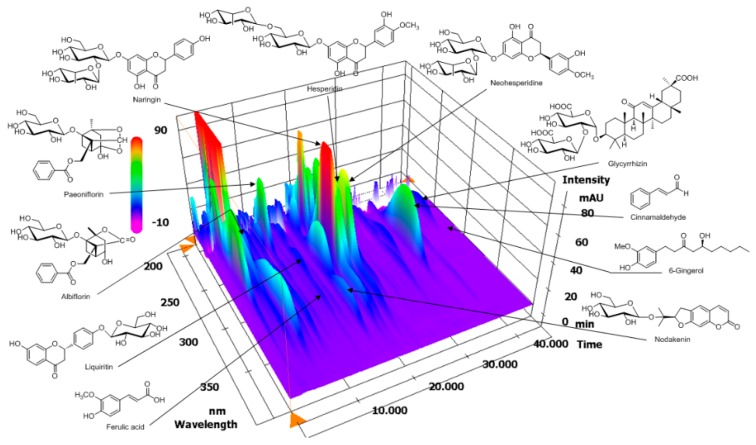
Three-dimensional chromatogram of OJS obtained using a high-performance liquid chromatography-photodiode array (HPLC-PDA).

The optimized HPLC–PDA method was applied for the simultaneous analysis of 11 marker ingredients in OJS. The 11 marker components were separated within 45 min. A representative three-dimensional HPLC chromatogram is shown in Figure 1. The retention times of albiflorin, paeoniflorin, liquiritin, ferulic acid, nodakenin, naringin, hesperidin, neohesperidin, cinnamaldehyde, glycyrrhizin, and 6-gingerol were 9.31, 10.65, 14.17, 14.96, 16.64, 18.86, 20.02, 21.74, 33.25, 41.42, and 42.34 min, respectively. The correlation coefficient of the calibration curves for the 11 compounds showed good linearity, ≥0.9997. The concentrations of the 11 compounds in lyophilized OJS sample were between 0.15 and 4.52 mg/g. Among these components, hesperidin, a marker compound of Citri unshii Pericarpium, was found to be the main compound in the OJS sample.

### 3.2. The Effect of OJS on Food Intake and Body Weight

There was no significant change in food intake each group. Body weight was significantly increased in the ApoE −/− mice group compared with the control group. The OJS group showed significantly increased body weight compared with the ApoE −/− mice group (Table 3).

### 3.3. The Effect of OJS on Lipid Parameters in ApoE −/− Mice

ApoE −/− mice group fed a Western diet showed increased levels in plasma triglyceride, total cholesterol, LDL/VLDL-cholesterol levels, and atherogenic index. However, these levels were significantly suppressed by treatment of OJS. The plasma levels of HDL-cholesterol levels also increased compared to those in the disease group by OJS (Table 3).

### 3.4. Effect of OJS on Vascular Dysfunction in ApoE −/− Mice

**Figure 2:**
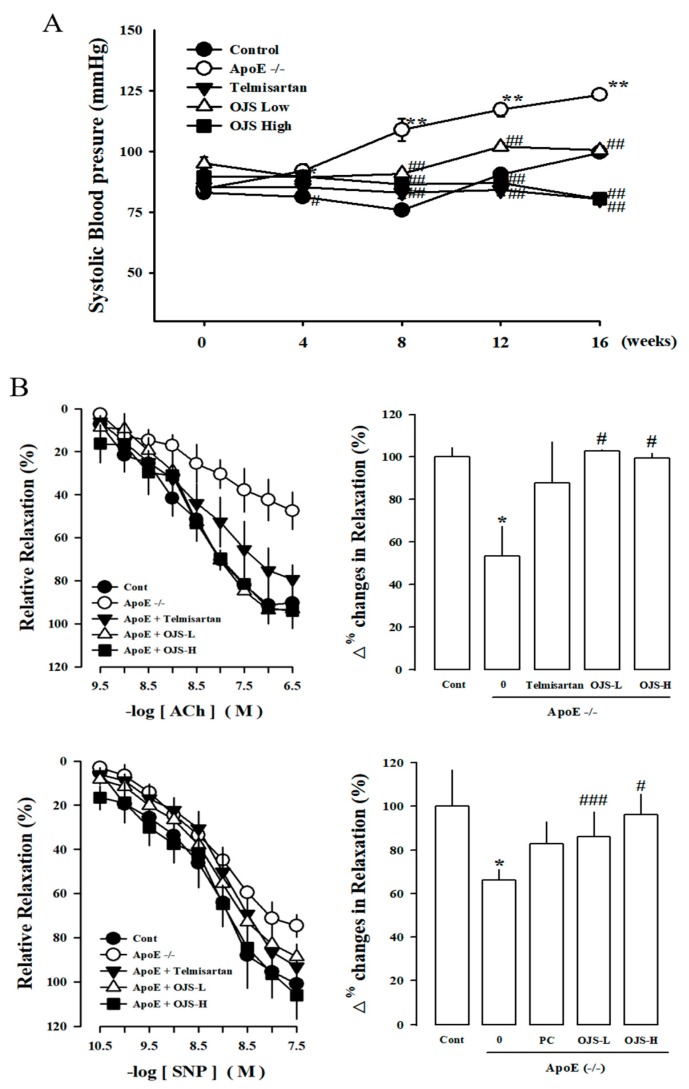
Effect of OJS on vasodilation in Apolipoprotein-E gene knockout (ApoE −/−) mice. The effect of OJS on systolic blood pressure in ApoE −/− mice (**A**). Cumulative concentration–response curves to acetylcholine (ACh) and the endothelium-dependent vasodilator, sodiumnitroprusside (SNP), in the arteries from experiment mice (**B**). PC, positive control; Values are expressed as mean ± S.E. (Standard Error) (*n* = 5). * *p* < 0.05, ** *p* < 0.01 vs. control group; ^#^ *p* < 0.05, ^##^ *p* < 0.01, ^###^ *p* < 0.001 vs. the ApoE −/− group.

The levels of SBP from all experimental groups were approximately 85–95 mmHg at the starting point of study. After four weeks, systolic blood pressure in the ApoE −/− group was significantly increased relative to that of the control (*p* < 0.05). After 8 weeks, systolic blood pressure in the ApoE −/− group was further increased (*p* < 0.01). However, in the OJS group, blood pressure was significantly decreased relative to that of the ApoE −/− group (Figure 2A). Vascular responses to ACh, an endothelium-dependent vasodilator (10^−10^–10^−6^ M), and SNP, an endothelium-independent vasodilator (SNP, 10^−11^–10^−7^ M), were measured in the carotid artery. Responses to ACh-induced relaxation of carotid artery rings were significantly decreased in the disease group compared to that in the control group. However, the impairment in vasorelaxation was remarkably decreased by treatment with OJS. (Figure 2B). 

### 3.5. Effect of OJS on Atherosclerotic Lesions in ApoE −/− Mice

**Figure 3:**
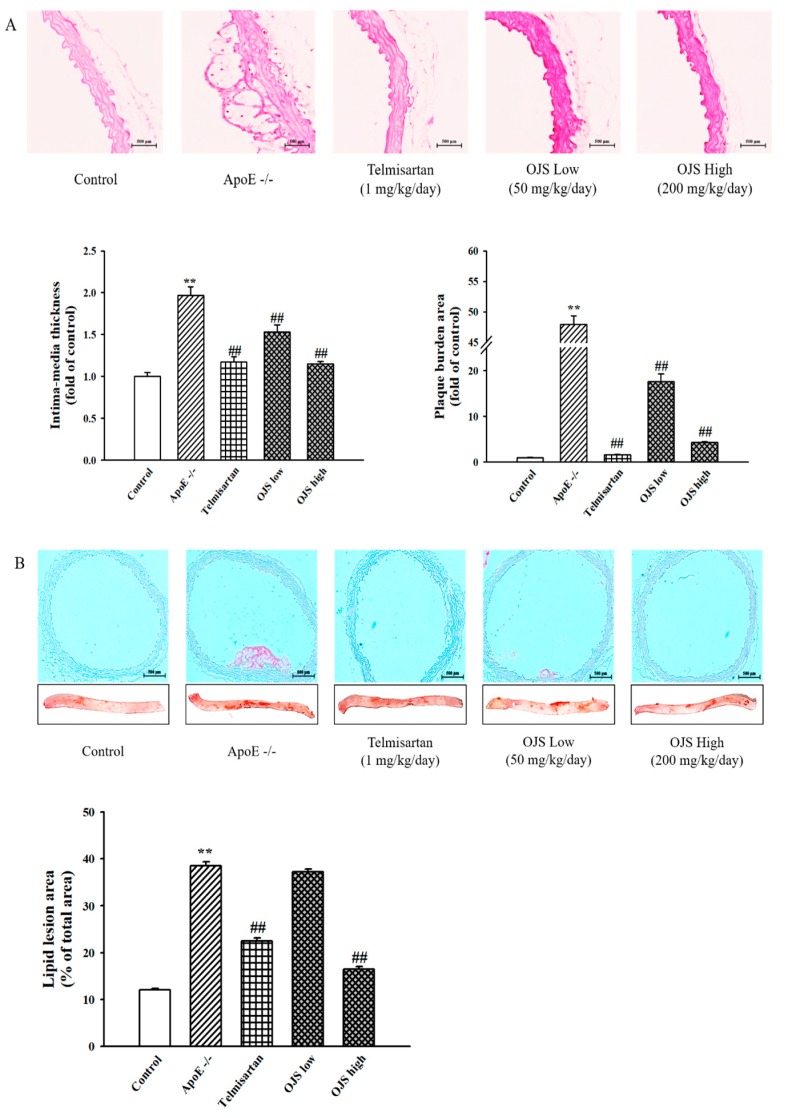
Effect of OJS on atherosclerotic lesion in ApoE −/− mice. Microscopic photographs of the aorta stained with hematoxylin and eosin (H&E) (**A**). Lipid lesion area staining in the aorta (100×). Thoracic and abdominal aorta from the indicated genotype were cut open with the luminal surface facing upward, and the inner aortic surface was stained with Oil Red O (**B**). Data are presented as means ± S.E. ** *p* < 0.01 vs. control group; ^##^ *p* < 0.01 vs. ApoE −/− group.

To investigate the effect of OJS on the morphology of the aorta, histological changes were observed by staining with H&E. Morphological staining showed that aortas of the ApoE −/− group increased in layer thickness, plaques, and inflammatory lesions compared to those of the control group (400×) (Figure 3A). However, OJS decreased the intima-media thickness in aortic sections and the plaque area. In addition, to confirm the inhibitory effects of OJS on lipid accumulation, Oil Red O staining was performed. In the control group, no atherosclerotic lesion in the aorta was detected. In the disease group, most of the lesions, identified as areas that stained red, were found at the aortic sinus; Oil Red O staining analysis demonstrated that aortic atherosclerotic lesions significantly increased in the ApoE −/− group compared with the control group. However, consistent with the change in the lipid profile, treatment with positive control (telmisartan) and OJS significantly inhibited the development of atherosclerosis (Figure 3B).

### 3.6. Effect of OJS on Vascular Inflammation in ApoE −/− Mice

**Figure 4:**
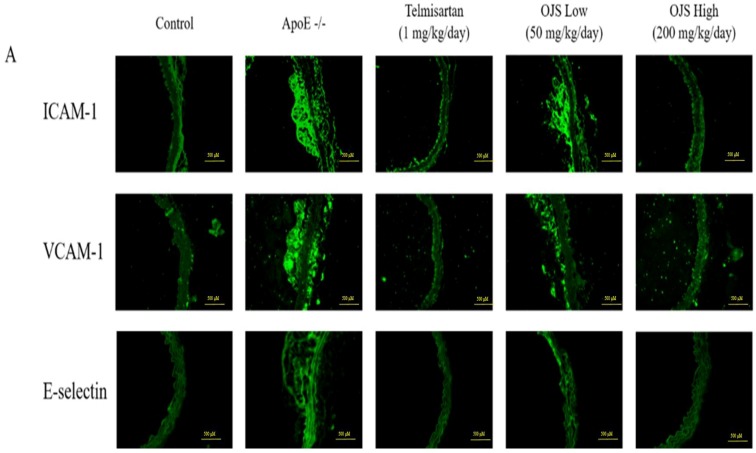

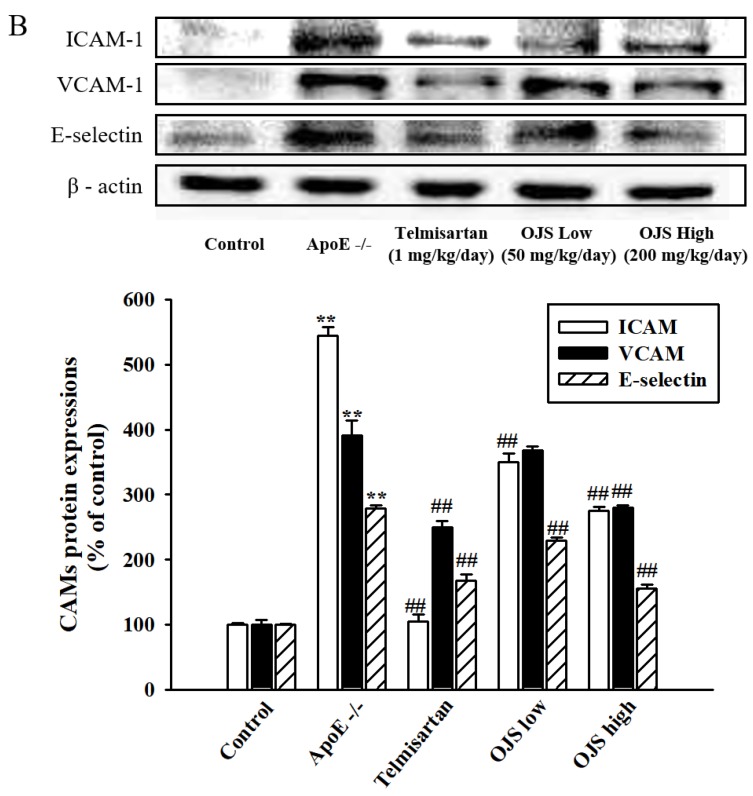
Effects of OJS on intracellular adhesion molecule-1 (ICAM-1), vascular cell adhesion molecule-1 (VCAM-1), and endothelial cell selectin (E-selectin) expression in the aortas of ApoE −/− mice (**A**). ICAM-1, VCAM-1, and E-selectin immunofluorescence in the aortas of ApoE −/− mice. Immunofluorescence staining of adhesion molecules in the aortas from the control, ApoE −/−, ApoE −/− mice treated with telmisartan groups, and ApoE −/− mice treated with OJS at low or high concentrations. Protein levels of the adhesion molecules determined by Western blot analysis (**B**). Data are presented as means ± S.E. ** *p* < 0.01 vs. control group; ^##^ *p* < 0.01 vs. ApoE −/− group.

**Figure 5:**
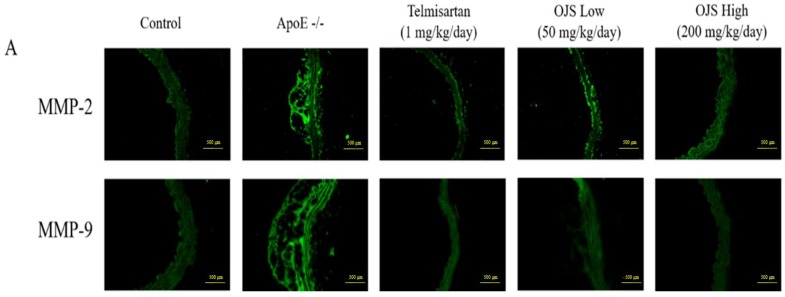

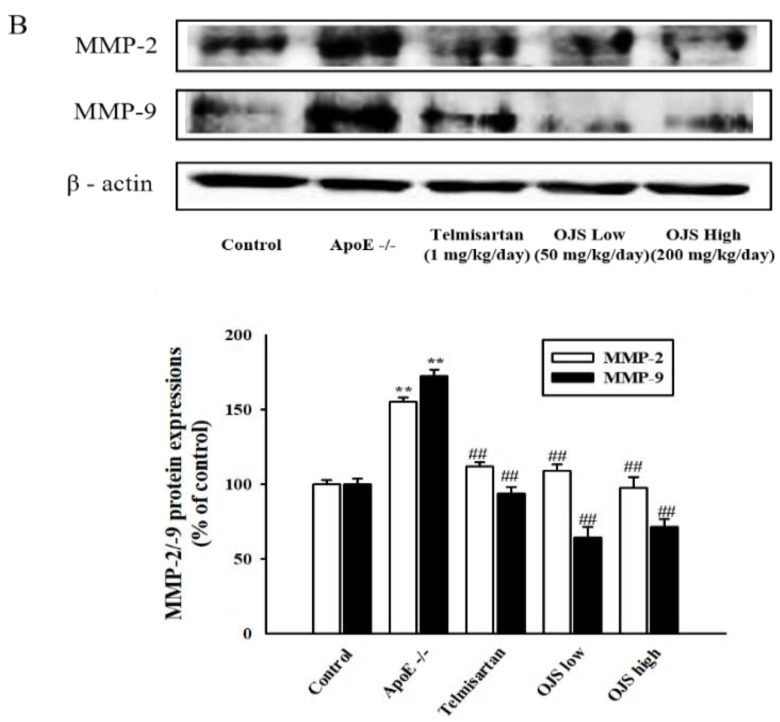
Effects of OJS on matrix metalloproteinases (MMP)-2/-9 expression in the aorta of ApoE −/− mice. MMP2, and MMP-9 immunofluorescence in the aorta of ApoE −/− mice (**A**). Immunofluorescence staining of MMP-2/-9 in the aorta from the control, ApoE −/−, and ApoE −/− mice treated with telmisartan groups and ApoE −/− mice treated with OJS at low or high concentrations. Protein levels of MMPs determined by Western blot analysis (**B**). Data are presented as means ± S.E. ** *p* < 0.01 vs. control group; ^##^ *p* < 0.01 vs. ApoE −/− group.

**Figure 6:**
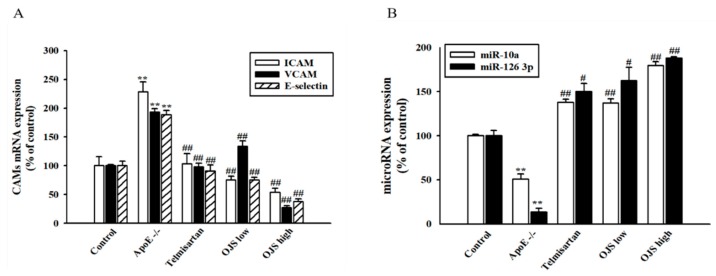
Effects of OJS on miR-10a and miR-126 3p expression in the aorta of ApoE −/− mice. mRNA expression of adhesion molecules determined by real-time Reverse Transcription-PCR (RT-qPCR) analysis (**A**). Levels of miRNA determined by real-time RT-qPCR (**B**). Data are presented as means ± S.E. ** *p* < 0.01 vs. control group; ^#^ *p* < 0.05, ^##^ *p* < 0.01 vs. ApoE −/− group.

Immunofluorescence was performed to determine the direct expression of adhesion molecules in the aortic wall. Expression of adhesion molecules such as ICAM-1, VCAM-1, and E-selectin increased in the disease group compared to that of the control. However, treatment with OJS significantly decreased the expression levels of ICAM-1, VCAM-1, and E-selectin (Figure 4A). The OJS group had significantly decreased levels of ICAM-1, VCAM-1, and E-selectin proteins compared to those of the ApoE −/− group (Figure 4B). The expression of MMP-2/-9 was increased in the ApoE −/− group (Figure 5A). Treatment with OJS also significantly decreased the expression levels of MMPs. Protein levels of MMP2/9 were increased in the disease group compared to those in the control group. Treatment with OJS significantly decreased the expression levels of MMP proteins compared to those in the disease group (Figure 5B). In Figure 6A, mRNA expression levels of ICAM-1, VCAM-1, and E-selectin were increased in the ApoE −/− group compared to the control group. OJS significantly suppressed mRNA expression levels. To confirm the effect of OJS on the mRNA expression of adhesion molecules in greater detail, miR10a and miR126-3p expression levels were determined. These microRNAs are known to regulate adhesion molecule expression and the IκB pathway. The expression levels for the miRNAs decreased in the disease group. However, treatment with OJS significantly increased the expression levels of these miRNAs (Figure 6B).

### 3.7. Effects of OJS on TNF-α-Induced Adhesion Molecules and MMPs Expression in HUVECs

**Figure 7:**
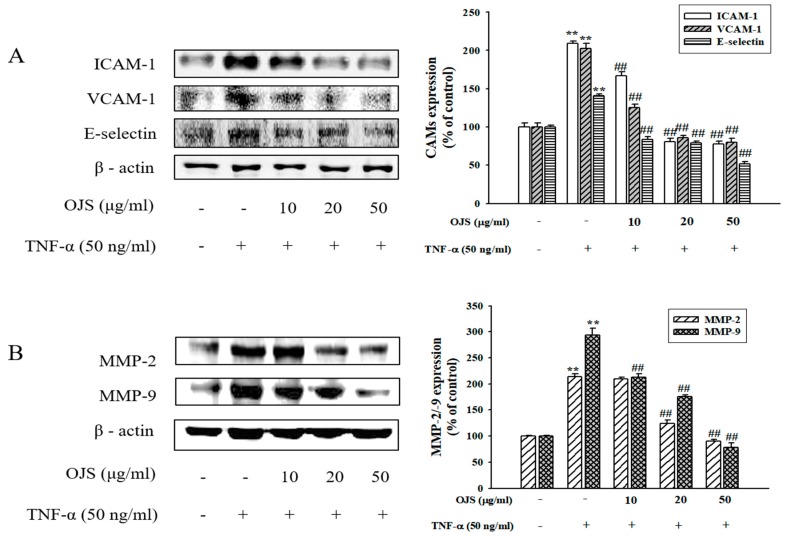
Effects of OJS on TNF-α-induced protein expressions of cell adhesion molecule and MMPs expression. Western blot analysis of ICAM-1, VCAM-1, and E-selectin expression. The blots are representative of three independent experiments and densitometric quantification of ICAM-1, VCAM-1, and E-selectin (**A**). Western blot analysis of MMP-2/-9 whole protein (**B**). Data are presented as means ± S.E. ** *p* < 0.01 vs. control; ^##^ *p* < 0.01 vs. TNF-α alone.

Treatment of OJS significantly inhibited the TNF-α-induced expression of ICAM-1, VCAM-1, and E-selectin (*p* < 0.05) (Figure 7A). Western blot analysis of cell lysates was used to confirm the effect of OJS on the expression of MMP-2/9 proteins in HUVECs. Pretreatment with OJS inhibited TNF-α-induced MMP2/9 protein expression (Figure 7B).

### 3.8. OJS Regulates the Akt/eNOS-NO Pathway in HUVECs

**Figure 8:**
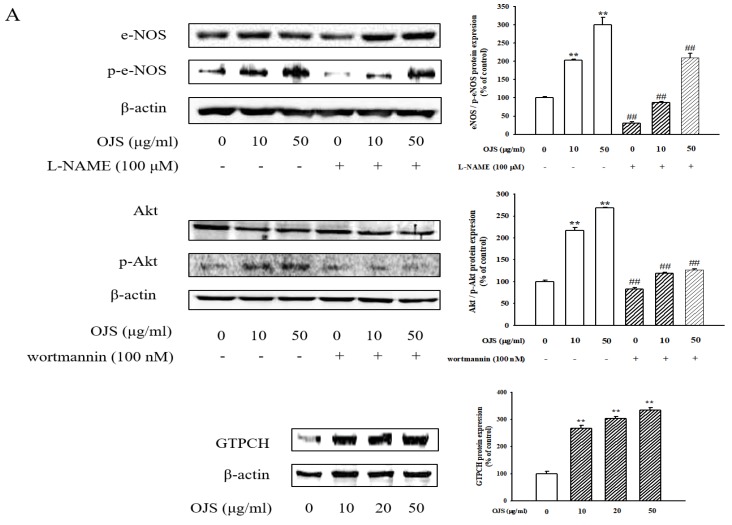

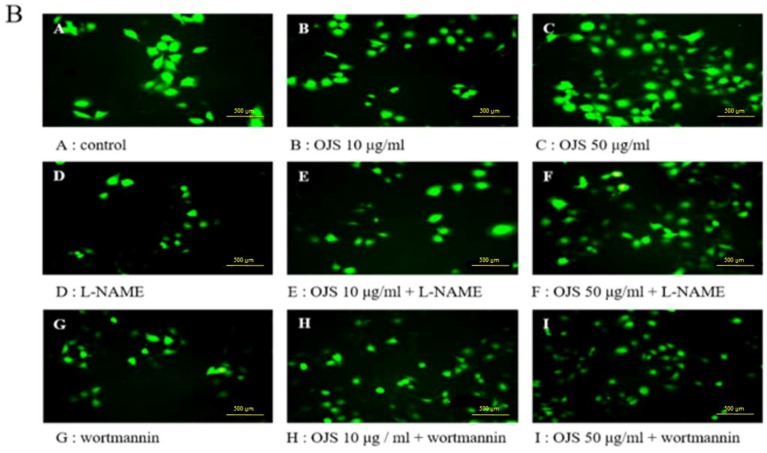

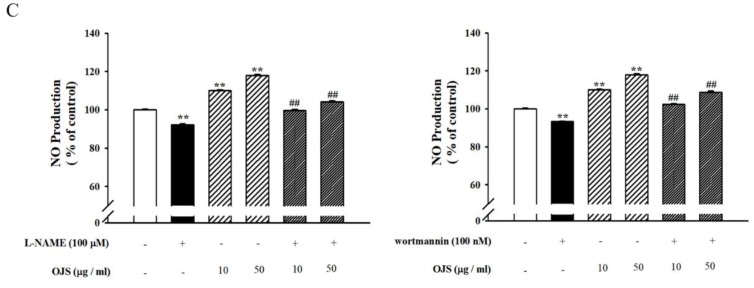
Effects of OJS on nitrite production. Protein expression of phosphorylated endothelial nitric oxide synthase (eNOS), phosphorylated protein kinase B (Akt), and GTP cyclohydrolase-1 (GTPCH) were analyzed by Western blotting (**A**). NO production was examined via fluorescence microscopy (original magnification ×100) (**B**). The effect of OJS on NO production was assayed as its stable reaction product nitrite by using the Griess reaction (**C**). Data are presented as means ± S.E. ** *p* < 0.01 vs. control, ^##^ *p* < 0.01 vs. OJS treatment. l-NAME, N(G)-nitro-l-arginine methyl ester.

Endothelial cells were treated with different doses of OJS for 30 min, and the degree of phosphorylation of eNOS and Akt was determined by Western blot analysis. As shown in Figure 8A, phosphorylation of eNOS and Akt increased following treatment with OJS in a dose-dependent manner. There were no significant differences in eNOS and Akt expression. OJS also increased the expression of GTPCH. These results suggest that OJS stimulates eNOS and Akt phosphorylation in HUVECs and regulates the eNOS coupling pathway by increasing the expression of GTPCH. Figure 8B shows that OJS treatment increased the production of NO in HUVECs in a dose-dependent manner. In addition, l-NAME (N(ω)-nitro-l-arginine methyl ester) and wortmannin as inhibitors of eNOS and Akt each inhibited OJS-induced production of NO (Figure 8C).

## 4. Discussion

OJS has been used to treat circulation disadvantage of qi (氣), blood (血), food (食), cold (寒), and congestion (痰). However, there are no reports regarding the protective effect of OJS against blood circulation disorders such as cardiovascular diseases. Here, we are the first to provide evidence indicating that OJS has an anti-atherogenic effect, improving vascular dysfunction in endothelial cells and Western-diet-fed ApoE −/− mice. 

OJS has an effect on plasma HDL-cholesterol levels, which is related to cardiovascular disease. HDL can remove cholesterol from macrophage foam cells and suppresses atherosclerotic lesions [24]. Triglycerides are an important biomarker of cardiovascular disease. In this study, blood glucose, systolic blood pressure, and lipid parameter levels were measured, and these levels were increased in Western-diet-fed ApoE −/− mice. Treatment with OJS significantly reversed these changes. These findings demonstrate that OJS may elicit a protective role against the initiation and development of atherosclerosis by improving lipid metabolism. Overall, the ApoE −/− groups (WTD-fed group) were found to weigh more than the control group (RD-fed group). However, it is confirmed that there is no change in the body weight of OJS-treated group. The endothelium can sense changes or abnormalities in blood flow and pressure. The vascular endothelium also plays an important role in the modulation of vascular tone [25]. From these results, it is clear that the mean SBP was higher in the ApoE −/− group. However, treatment with telmisartan, both low and high dosages of OJS, significantly decreased mean SBP. This study also evaluated the effects of OJS on histological changes by examining the aorta using oil red O and H&E staining. Our results indicate that OJS administration significantly resolved atherosclerotic plaque formation in the aorta. In addition, the ApoE −/− group also showed endothelial dysfunction, as evidenced by decreases in ACh- and SNP-induced vascular tone. These findings suggest that the hypotensive effect of OJS is mediated by an endothelium-dependent NO/cGMP pathway. 

The present study revealed that OJS can regulate the early and advanced stages of atherosclerotic process, which are linked closely the inflammatory response of blood vessels to injury caused by atherosclerotic plaques, which can lead to cardiovascular diseases [26]. miR-126 has been reported to reduce the expression of ICAM-1, VCAM-1, and E-selectin by directly targeting the 3′ untranslated region (3′UTR) of these genes [6,7]. miR-10a targeted two proteins MAP3K7 (TAK1) that regulate IκB degradation [8]. Therefore, the expression of miR-10a and miR-126 3p were investigated. The results showed that the expression levels were decreased in the Western-diet-fed ApoE −/− mice. However, OJS increased the expression of these miRNAs. Therefore, these results suggest that OJS has an inhibitory effect on the adhesion molecule pathway and IκB degradation by regulating the expression of miRNA. MMPs also damage the vascular extracellular matrix, resulting in weakening and dilatation of the aortic wall, which is a hallmark of vascular inflammation [27]. Therefore, the inhibition of MMP2/9 could be beneficial in the treatment of atherosclerosis. The results of immunofluorescence and Western blot analysis revealed that pretreatment with OJS suppressed the expression of MMP2/9. These results indicate that OJS has an inhibitory effect on MMP-2 and MMP-9 expression levels in ApoE −/− mice.

Cytokines-induced adhesion molecules such as ICAM-1, VCAM-1, and E-selectin are well known inflammatory markers [28]. Therefore, the present study was examined whether OJS has an inhibitory effect on TNF-α-stimulated HUVECs by inhibiting the protein expression of adhesion molecules. The results suggested that OJS has an inhibitory effect on TNF-α-induced vascular inflammation in endothelial cells by suppressing the expression of those adhesion molecules. Endothelial dysfunction, characterized by decreased production of NO, is an early and key mediator that links obesity and cardiovascular diseases [29]. Akt downstream of PI3K, is also thought to be an important factor for cell survival. In endothelial cells, Akt activation has been reported to promote cell survival [14]. Importantly, several clinical studies have demonstrated the beneficial effects of BH4, which is a required cofactor for the synthesis of NO, in patients with cardiovascular risk factors, such as hypercholesterolemia, smoking, hypertension, and diabetes or coronary artery disease [30]. Furthermore, endothelial cell BH4 synthesis by GTPCH is necessary for physiological eNOS function, and previous studies of BH4 biosynthesis have used systemic pharmacological inhibitors of GTPCH [31]. Data from the current study indicate that OJS promoted NO production. This study also examined the role of the PI3K/Akt pathway and phosphorylation of eNOS in the anti-inflammatory effect of OJS. eNOS and Akt phosphorylation were also increased by OJS. These results provide strong evidence that OJS elicits an anti-inflammatory effect via the PI3K/Akt-dependent eNOS pathway. In addition, eNOS coupling is a well-known defense mechanism against vascular disease via regulation of NO production [32]. The expression of the principal factor that regulates eNOS coupling, GTPCH, was increased by OJS. This result indicates that OJS plays a protective role in vascular dysfunction by regulating eNOS coupling. Therefore, further studies on the effect of OJS on expression of BH4 in HUVEC should be performed. There is a previous study to confirm the improvement of OJS in liver inflammation [19], and the present study similarly confirmed the improvement of atherosclerosis in OJS by suppressing the expression of atherogenic and inflammatory factors. Therefore, further studies to determine which of the 17 components in OJS have the effect of relieving atherosclerosis are also needed. 

## 5. Conclusions

**Figure 9:**
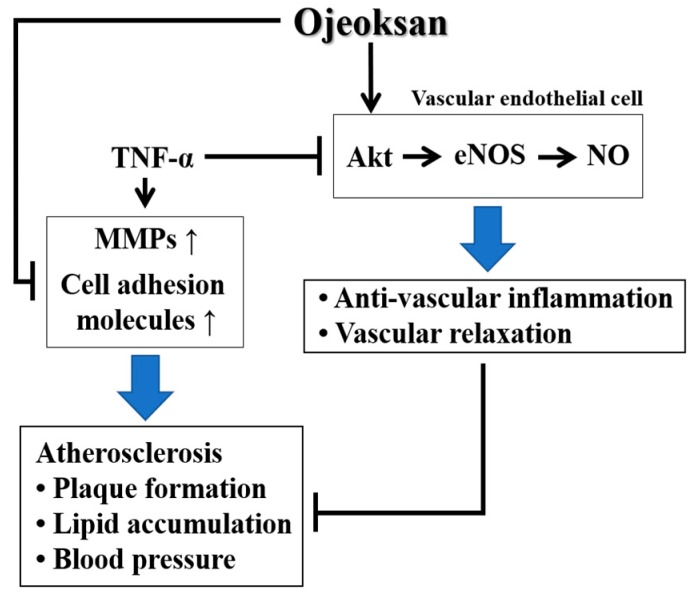
Schematic diagram of the effects of OJS in atherosclerosis.

OJS treatment markedly lowered vascular dysfunction and inflammatory processes. OJS treatment not only ameliorated impairment of vascular dysfunction and metabolic abnormalities but also markedly lowered blood pressure, as well as vascular inflammatory processes in ApoE KO mice and HUVECs (Figure 9). To the best of our knowledge, these findings provide the first evidence to support the therapeutic efficacy of OJS in preventing the development of both early and advanced atherosclerosis.

## Figures and Tables

**Table 1:** Decoction of Ojeoksan (OJS).

Latin Name	Scientific Name	Amount (g)	Origin
Atractylodis Rhizoma	*Atractylodes lancea* DC	7.5	China
Ephedrae Herba	*Ephedra sinica* Stapf	3.7	China
Citri unshii Percarpium	*Citrus reticulata* Blanco	3.7	Jeju, Korea
Magnoliae Cortex	*Magnolia officinalis* Rehder & E.H. Wilson	3.0	China
Platycodi Radix	*Platycodon grandiflorus* (Jacq.) A. DC	3.0	Yeongcheon, Korea
Aurantii Fructus Immaturus	*Citrus aurantium* L	3.0	China
Angelicae Gigantis Radix	*Angelica gigas* Nakai	3.0	Pyeongchang, Korea
Zingiberis Rhizoma	*Zingiber officinale* Roscoe	3.0	Yeongcheon, Korea
Paeoniae Radix	*Paeonia lactiflora* Pall	3.0	Hwasun, Korea
Poria Sclerotium	*Wolfiporia* extensa	3.0	Yeongcheon, Korea
Angelicae Dahuricae Radix	*Angelica dahurica* (Hoffm.) Benth. & Hook.f. ex Franch. & Sav	2.6	Yeongcheon, Korea
Cnidii Rhizoma	*Ligusticum officinale* (Makino) Kitag	2.6	Yeongcheon, Korea
Pinelliae Tuber	*Pinellia ternata* (Thunb.) Ten. ex Breitenb	2.6	China
Cinnamomi Cortex	*Cinnamomum cassia* (L.) J. Presl	2.6	Vietnam
Glycyrrhizae Radix et Rhizoma	*Glycyrrhiza uralensis* Fisch	2.2	China
Zingiberis Rhizoma recens	*Zingiber officinale* Roscoe	3.7	Hanam, Korea
Allii Fistulosi Bulbus	*Allium fistulosum* L	3.7	Hanam, Korea

**Table 2:** Diagrams of concerning composition in regular diets (RD) and Western diets (WTD) for experimental mice.

Product	D12450B		D12079B	
	gm %	kcal %	gm %	kcal %
Protein	19.2	20	20	17
Carbohydrate	67.3	70	50	43
Fat	4.3	10	21	40
Total		100		100
kcal/gm	3.85		4.7	
**Ingredient**	**gm**	**kcal**	**gm**	**kcal**
Casein, 80 Mesh	200		195	
dl-Methionine			3	
l-Cystine	3			
Corn Starch	315		50	
Maltodextrin 10	35		100	
Sucrose	350		341	
cellulose	50		50	
Milk Fat, Anhydrous			200	
Corn oil			10	
Soybean oil	25			
Lard	20			
Mineral Mix S10026	10		35	
Dicalcium Phosphate	13			
Calcium Carbonate	5.5		4	
Potassium Citrate, 1 H_2_O	16.5			
Vitamin Mix V10001	10		10	
Choline Bitartrate	2		2	
Cholesterol, USP	0.05		1.5	
FD&C Yellow Dye #5				
**Total**	**1055.05**	**4057**	**1001.54**	**4686**

Unitied Statees Pharmacopeia (USP); Federal Food, Drug, and Cosmetic Act (FD&C).

**Table 3:** Effect of OJS treatment on plasma biomarker levels in Apolipoprotein-E gene knockout (ApoE −/−) mice.

	Food Intake (g)	Body Weight (g)	T-Cho (mg/dL)	TG (mg/dL)	LDL/VLDL-Cho (mg/dL)	HDL-Cho (mg/dL)	Atherogenic Index
**Control**	3.90 ± 0.12	29.26 ± 0.92	64.69 ± 2.89	78.71 ± 1.25	27.51 ± 3.31	46.91 ± 1.04	0.38 ± 0.06
**ApoE −/−**	3.82 ± 0.42	33.62 ± 1.32 **	271.78 ± 9.79 **	122.30 ± 1.90 **	128.58 ± 5.03 **	20.02 ± 1.10 **	12.69 ± 1.12 **
**Telmisartan**	4.14 ± 0.46	35.68 ± 0.86	100.52 ± 1.12 ^##^	90.28 ± 1.38 ^##^	87.20 ± 1.26 ^##^	32.96 ± 1.82 ^##^	2.07 ± 1.12 ^##^
**OJS low**	3.97 ± 0.07	37.14 ± 0.41	179.55 ± 2.42 ^##^	104.28 ± 0.76 ^##^	107.20 ± 0.82 ^#^	22.62 ± 1.70	7.01 ± 0.54 ^##^
**OJS high**	3.72 ± 0.23	35.48 ± 1.25 ^#^	122.73 ± 4.84 ^##^	90.06 ± 1.46 ^##^	79.43 ± 2.88 ^##^	30.21 ± 2.23 ^#^	3.11 ± 0.33 ^##^

The control group (C57BL6 mice + regular diet + Distilled Water (DW)), ApoE −/− group (ApoE −/− + Western diet + DW), telmisartan group (ApoE −/− + Western diet + telmisartan 1 mg/kg/day), OJS low group (ApoE −/− + Western diet + OJS 50 mg/kg/day), OJS high group (ApoE −/− + Western diet + OJS 200 mg/kg/day). ApoE −/−, apolipoprotein E knockout; T-Cho, total cholesterol; TG, triglyceride; LDL/VLDL-Cho, low-density lipoprotein/very low-density lipoprotein-cholesterol; HDL-Cho, high-density lipoprotein-cholesterol. Data are mean ± S.E. values (*n* = 12). (** *p* < 0.01 vs. control and ^#^ *p* < 0.05, ^##^ *p* < 0.01 vs. ApoE −/−).
